# Color Assessments and Glycolysis of Cetylpyridinium Chloride-Containing Aqueous Solutions and Commercial Mouthwashes

**DOI:** 10.3390/mps9010010

**Published:** 2026-01-11

**Authors:** Robert L. Karlinsey, Tamara R. Karlinsey

**Affiliations:** Custom Dental Formulations, LLC, 1291 Airport Parkway, Suite 400, Greenwood, IN 46143, USA

**Keywords:** bioavailability, pH, CPC, antimicrobial, oral care, preventive dentistry, plaque, gingivitis

## Abstract

**Background**: Effective cetylpyridinium chloride (CPC)-based mouthwashes critically depend on maintaining maximum levels of bioavailable CPC to deliver optimum antimicrobial benefits. While this is traditionally assessed using cellulose-based methods, from economic and efficiency perspectives, there remains a need to identify other potential methods of assessing bioavailable CPC. Here, we explored whether quaternary ammonium compound (QAC) test strips are sensitive to CPC-based formulations, and if so, whether there might exist a possible correlation with glycolysis outcomes. **Methods**: Quantitative color parameters were obtained using spectrophotometric assessments of QAC test strips immersed in simple CPC solutions and eight commercial CPC-based mouthwashes available in the USA. Then, using our established glycolysis model, we assessed the glycolytic response of both the simple CPC solutions and commercial CPC-based mouthwashes, and compared these data sets. **Results**: Significant differences (*p* < 0.05) among the CPC simple solutions were found. Importantly, spectrophotometric assessments and glycolysis trials produced good correlations. Evaluations of the commercial mouthwashes further underlined this correlation, even though those that comprise zinc salts may impact QAC-based color. **Conclusions**: Based on these results, we believe the use of QAC test strips provides an attractive option to formulators and brands specializing in the development and/or testing of CPC-based oral care formulations.

## 1. Introduction

The United States Food and Drug Administration (FDA) outlines specific requirements in the over-the-counter (OTC) antiplaque/antigingivitis monograph for oral care formulations that utilize cetylpyridinium chloride (CPC) [1,2]. If a formulation comprises CPC, the monograph stipulates that a minimum level of bioavailable CPC must be available. The fact this requirement exists speaks to the sensitivity of CPC to other ingredients that may encumber its performance.

The traditional method of measuring bioavailable CPC, which was originally based on alcohol-based formulations, is based on the cellulose disk retention assay method (DRA) [3,4,5]. This method involves dispensing aliquots of a given CPC solution onto a moist stack of cellulose discs, then centrifuging them, and then measuring the level of CPC in the supernatant via HPLC with UV detection [4]. There are two major drawbacks with this technique [5]. First, although experimental timings, necessary buffering, and the need for the specialized equipment to perform this assessment may present some challenges, ultimately, the cellulosic discs can only adsorb so much CPC (which may bear on the upper limit available in a given formulation)—aspects that might be heavily influenced by the centrifugation speeds, quality of the discs, and skill of the technician. Second, the DRA method primarily responds to the primary monomer-like forms of CPC, which manifest a significantly large dipole due to the long fatty chain terminated by the nitrogen-based cationic head group. Because standard CPC mouthwashes have loading levels higher (e.g., between 450 and 1000 ppm CPC) than the critical micelle concentration (e.g., ~289 ppm CPC) [6], higher-order structures or geometries, such as micelles, vesicles, and polymer-like chains of CPC will exist [5,6,7,8,9]. As the dipoles from these structures are not as prominent as the monomer-like CPC structure, the DRA method can underestimate the level of bioactive CPC of a given mouthwash formulation, especially since salts and polymers (including polyethylene glycol) can impact micelle and other higher-order formations [5,6,7,8,9]. This is one reason that alcohol, which disrupts higher-order structures, was historically used in combination with CPC in mouthwash formulations [2,5].

Confirmation of CPC bioactivity is of paramount importance, and this likely includes contributions from both monomer-like molecules and higher-order structures [5]. Aside from glycolysis (which may or may not agree with DRA assessments depending on the formulation [3,4,5], nuclear magnetic resonance (NMR) has been employed to help characterize CPC-based systems [5]. Still, glycolysis remains a powerful method due to its direct insight into the response of microbial species, which is highly relevant for formulations targeting plaque and/or gingivitis [3,4,10,11]. To that end, we have established a versatile mixed-microbial species model that provides critical insight into how a given formulation might perform and we routinely use this method in our contract formulation and testing endeavors [11].

To minimize uncertainty in potential efficacy of a given formulation (which will comprise other ingredients that may or may not influence CPC structure and availability [1,2,3,4,5,6,7,8,9,10,11]), formulators and researchers need access to screening methods other than DRA and NMR. However, and to the best of our knowledge, the application of quaternary ammonium compound (QAC) test strips to aid in the development or evaluation of CPC-based systems has not been explored. In contrast to the cellulose disc methodology, QAC test strips are inexpensive, widely available, and routinely used to assess potency of antiseptic or cleaning agents where microbial contamination is of concern [12,13,14]. QACs are sensitive due to the reaction of the QAC (e.g., CPC) with an organic dye molecule, such as bromocresol green or bromophenol blue [15,16,17,18,19]. These reactions, which are based on the reactivity of the dye molecule in the presence of an appropriate buffer, are based on ion-pair formations evolved when QAC contacts the dye. A reactive QAC indicates the positively charged molecule has not been otherwise neutralized by components of a given formulation (e.g., surfactants or negatively charged ions) and is therefore able to readily react with the negatively charged dye molecule. In contrast to the DRA method, these strips do not rely on large dipoles to detect CPC, so detection is likely based on several structural confirmations.

Under acidic conditions, for instance, the dye will be anionic form and will produce a yellow color. There are at least two conditions for which the color of the dye molecule will change from yellow to green (or blue):

1. When the pH is increased (e.g., through contact with a base, such as sodium hydroxide), or;
2. When the dye molecule reacts with QACs, such as cetylpyridinium chloride.

It is through the #2 reaction for which the QAC test strips work. For this reaction to proceed by the sole reaction of the QAC compound (e.g., CPC), there needs to be negligible change in pH to eliminate acid–base reactions. This is accomplished with sufficiently strong buffering. An example of such a buffer in the presence of bromophenol blue is given by Conklin [15] in Table 1.

The recipe in Table 1 reveals the acidic nature of the buffer, which is used to avoid color changes due to pH. The reaction of the positively charged QAC will then shift the color from yellow to either green or blue, depending on the type and concentration of the dye used (e.g., bromocresol green and bromophenol blue, respectively). This color change is sometimes to referred to as the ‘pH indicator error’ [19,20]. Ergo, for a given CPC concentration (e.g., 0.07%), CPC bioavailability is highest when the CPC–dye interaction on the test strip is least yellow (i.e., more green or blue).

The purpose of this research is to identify whether QAC test strips are sensitive to CPC-based formulations, and if so, whether there might exist a possible correlation with glycolysis outcomes. The null hypothesis would be a lack of any correlation between the QAC test strips and glycolysis outcomes. In these experiments, we utilized a spectrophotometer to generate quantitative color parameters from commercially available QAC test strips immersed in simple CPC solutions or commercial CPC-based mouthwashes. Using our glycolysis model [11], we then assessed the glycolytic response of both simple CPC solutions and commercial CPC-based mouthwashes. These data sets were then compared, resulting in strong correlations between bioavailable CPC via QAC test strips and glycolytic activity. Based on these results, we believe the use of QAC test strips provides an attractive option to formulators and brands specializing in the development and/or testing of CPC-based oral care formulations.

## 2. Materials and Methods

Reagents, including CPC, were sourced from Fisher Scientific (Hampton, NH, USA). Solutions with distilled water (DIW) were made using calibrated pipettes and all containers and final solutions were sterilized accordingly.

The following details have been stated previously [11] and are given here for completeness: Sterilization (Moosoo Model No. GT606-M10) was performed on all relevant labware, solutions, and media to ensure only plaque-derived microbial responses. A Fisherbrand Accumet AB315 pH/mV meter fitted with a Mettler Toledo LE422 pH microelectrode were used to measure pH of all solutions and media. A Barnstead/Thermolyne Cimarec^®^ heat/stir plate and BT Lab Systems multi-position stir plate were used to magnetically agitate solutions and media using Teflon-coated stir bars [11].

Commercial OTC CPC-containing mouthwashes were purchased from Amazon.com (Seattle, WA, USA) and are detailed, along with measured pH, in Table 2. For the SmartMouth™ mouthwash, we mixed both solutions (1:1) according to manufacturer’s instructions: each compartment dispensed into the supplied cup manifesting a measurement line. In Table 2, ‘Other Ingredients’ refers to all the ingredients listed as ‘inactive ingredients’ on the manufacturers’ labels. As the negative control, we used 100% DIW and also refers to 0.0% CPC. For measurement of CPC, QAC test strips (Bartovation, West Harrison, NY, USA) were also sourced from Amazon.com (accessed 21 July 2025).

### 2.1. QAC Test Strip Immersions and Color Determinations

Spectrophotometric color assessments of L*, a*, and b* were made using a high-resolution (0.01) portable spectrophotometer equipped with a silicon photodiode array (VTSYIQI, Ser. No. CMDA9X5054). The numerical readings for these parameters correspond to the following color axes:

- Lightness: L* spans between 0 (black) and 100 (white).
- Green–Red: a* spans between more negative (green) and more positive (red).
- Yellow–Blue: b* spans between more negative (blueness) and more positive (yellow).

Due to the size of the QAC color squares, we used the 3 mm aperture to minimize extraneous light and background colors. Calibrations were made on both white and black substrates, and then measurements were made on QAC color squares accordingly. Each measurement produced L*, a*, and b* values and this was repeated in triplicate. The color space corresponding to L*, a*, and b* (LAB) is based on the International Commission on Illumination (CIE) [21]. The perceived change in color (*ΔE*) relative to a dry QAC strip (i.e., no immersion in water or any other solution) was calculated according to Equation (1) [21]:

(1) $$\Delta E=\sqrt{{\left(L_{2}^{*}-L_{1}^{*}\right)}^{2}+{\left(a_{2}^{*}-a_{1}^{*}\right)}^{2}{+\left(b_{2}^{*}-b_{1}^{*}\right)}^{2}},$$

where $L_{1}^{*}$, $a_{1}^{*}$, and $b_{1}^{*}$ correspond to the color of the reference (yellow 0 ppm) QAC strip. QAC test strips were immersed for two seconds in the test solution (simply CPC solution or commercial mouthwash) and then immediately measured with the spectrophotometer in triplicate.

### 2.2. Plaque Harvesting and Propagation

As described previously [11], the experimental procedure for this in vitro study utilized pooled human plaque donated from both authors (R.L.K. and T.R.K.) with caries experience but in present sound oral and systemic health. Each provided plaque samples after refraining from food, drink (except for water), and oral hygiene for at least 10 h overnight prior to collection as described previously [11]. Sterile cotton swabs were used to harvest the plaque (using a wiping technique from molar-to-incisor) in the morning at the buccal and lingual gingival margins in each quadrant of the mouth [11]. Each quadrant’s swab was then placed into a 7 mL tube containing 1.5 mL of 6% tryptic soy broth (TSB) and vortexed for 10 s. Afterwards, each vial’s contents were dispensed into a 20 mL sterile vessel with extracted swabs disposed of in biohazard bags. Each participant provided four vials, resulting in 6 mL of plaque–TSB mixture, which were immediately pooled, resulting in 12 mL of plaque-TSB fluid [11].

These plaque sample and growth techniques are based on published reports [4,10,11]. Each propagation progressed overnight (to at least 18 h) and produced high-density, malodorous media with pH near 4.4. Agar plating (not shown but available upon request) revealed the presence of mixed-microbial species, including (at minimum) lactobacilli, streptococci, Prevotella, and Bacteroides species.

### 2.3. Glycolysis Procedure and Calculation

The procedure for glycolysis has been slightly modified from our initial procedure and is given as follows [11]. On the morning of evaluation, aliquots of the propagated plaque were extracted and mixed 50:50 with a sterile 0.3% TSB + 0.5% sucrose solution [22]. The pH was then measured (~4.5) and the standardized to neutral (e.g., 7.0) using 1M NaOH. Then, 1 mL aliquots of this standardized plaque was combined with 17.5 µL of treatment (i.e., CPC solution or commercial mouthwash) in a sterilized 1.5 mL Eppendorf tube. Each vial was then vortexed at high-speed for 10 s and then baseline pH measurements were made. Two replicates were used per treatment group. Samples were then loaded into an Eppendorf 5384 F1.5 Thermomixer (Fisher Scientific, Waltham, MA, USA) set at 37 °C with 1200 rpm agitation and processed up to six hours, with periodic pH measurements made at the two-hour and four-hour timepoints.

In general, there are various ways to present glycolysis data generated from PGRM evaluations including simple differences, area-under-curves, lineshape fittings, percentages, and so forth [10,11,22]. We chose to calculate percent changes based on pH measurements made at baseline (*pH_Base_*) and up to six hours (*pH*_6*hrs*_) of thermomixing, and the mean change in pH at these two timepoints were determined according to Equation (2):

(2) $$\%\;Change\;in\;pH=\left(\frac{{pH}_{6hrs}-{pH}_{Base}}{{pH}_{Base}}\right)\times 100.$$

### 2.4. Data Sets and Statistical Analyses

Three replicates for the color measurement were assessed for each treatment group for L*, a*, and b*. Each pH measurement was performed in duplicate, and for a given treatment there were four measurements, as previously reported [11]: at baseline, two hours, four hours, and six hours. Descriptive statistics, including means, standard deviations (SD), and standard errors of the mean (SEM) were determined for each type of measurement. Treatments were then assessed for significance using a one-way analysis of variance (ANOVA) model using Sigma Plot version 14.5 (Systat Software). When significant differences were detected (*p* < 0.05), means were further evaluated pairwise using the Student–Newman–Keuls (SNK) test to identify where differences existed [11]. All data analyses (including Pearson correlation coefficient calculations) were determined using Excel version 365 (Microsoft, Seattle, WA, USA) and Microcal Origin version 6.0 (OriginLab Corporation, Northampton, MA, USA) software.

## 3. Results

The results discussed below include visual assessments of the QAC test strips, spectrophotometer measurements of the QAC test strips, and then separately performed glycolysis experiments probing the action of the simple CPC solution or commercial mouthwash.

### 3.1. Visual Assessment of QAC Test Strips Immersed in Simple CPC Solutions

**Figure 1:**
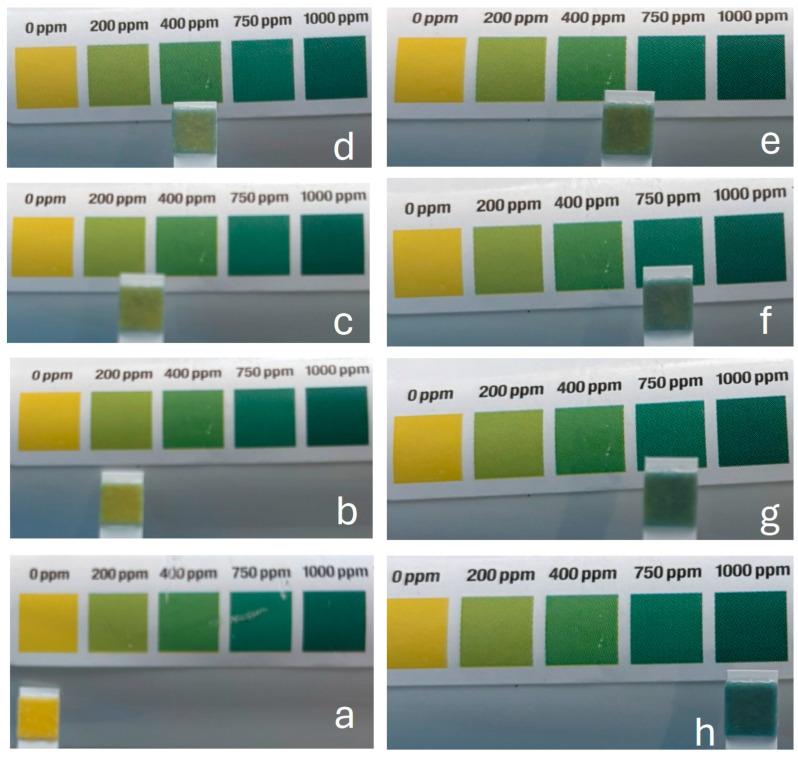
Colors of QAC test strips immersed in 0 (**a**), 200 (**b**), 300 (**c**), 400 (**d**), 500 (**e**), 700 (**f**), 750 (**g**), and 1000 (**h**) ppm CPC aqueous solutions. Colors range from yellow (0 ppm CPC) to dark green (1000 ppm CPC).

Qualitative responses of QAC test strips to brief immersions in different concentrations of CPC are shown in Figure 1. Beginning in the bottom left-hand image and rotating clockwise, the color of the QAC strip progresses from yellow (with no CPC) to dark green (1000 ppm CPC). Visually, there is little difference between 700 and 750 ppm CPC, which might be expected based on the sensitivity of the QAC strip, which is not designed to detect these differences. Similarly, the differences between 400 ppm and 750 ppm spans a significant range, though visually, this is challenging to resolve, especially among 500, 700, and 750 ppm CPC.

### 3.2. Spectrophotometry of QAC Test Strips Immersed in Simple CPC Solutions

Quantitative CIELAB color assessments of QAC strips immersed in simple CPC solutions are summarized in Table 3. In the table, L*, a*, and b* were measured and then input in Equation (1) to determine *ΔE* relative to the 0 ppm square on the QAC strip. This yellow square produced mean (standard deviation) L*, a*, and b* values of 89.56 (0.18), −0.82 (0.06), and 9.37 (0.41). The visual yellow color corresponds well to the very positive b* value, which indicates ‘more yellow’. The L* is inherently influenced by the label material, which through its glossy appearance contributes to the lightness. The a* value slightly favors the green color for the a* color axis.

Immersion of the QAC strips into distilled water (0 ppm CPC) and seven concentrations of CPC reveal distinct changes in L*, a*, and b*. With respect to L*, the lightness steadily diminishes with increasing CPC content (from 86.60 to 82.86). This is similar to but to a greater extent for the b* parameter, where the value ranges from 9.25 (‘more yellow’) for 0.0% CPC down to 1.28 (‘less yellow, more blue’) for 0.1% CPC. In contrast, a* remains relatively negative over the range in CPC content, indicating a ‘more green’ profile, which certainly reflects the visual coloring on the QAC at higher concentrations. When combined with b* and L*, the resulting perceived color difference, *ΔE*, is calculated and is listed in the fifth column. The largest color difference is observed for 0.1% CPC, which is significantly different than the other concentrations. Significant differences were also observed between 0.075% and 0.07% CPC, despite both having similar visual colors. At low CPC content, trends showed differences among 0.0%, 0.02%, and 0.03% CPC.

### 3.3. Glycolytic Responses from Simple CPC Solutions

**Figure 2:**
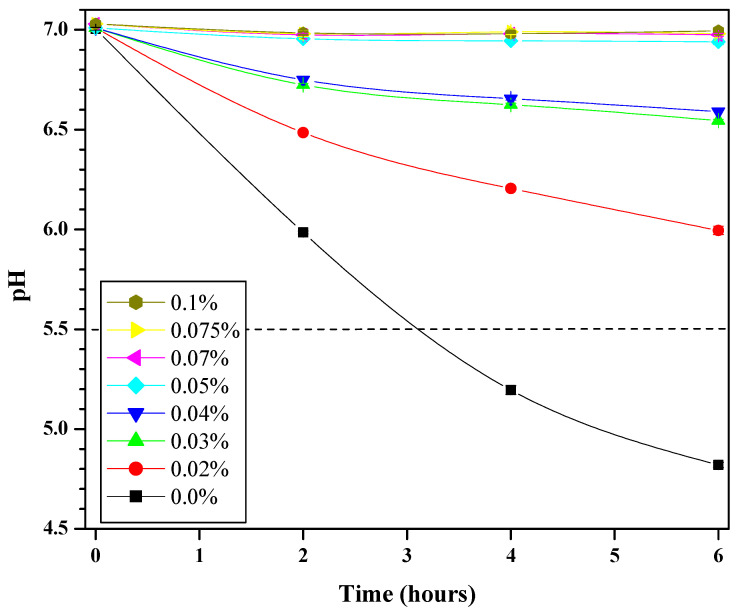
Mean (SD) glycolytic responses (as measured via pH in duplicate) produced from harvested human plaque treated with 0.0% (black squares, line), 0.02% (red sphere, line), 0.03% (green triangle, line), 0.04% (navy blue upside-down triangle, line), 0.05% (cyan diamond, line), 0.07% (magenta left-pointing triangle, line), 0.075% (yellow right-pointing triangle, line), or 0.1% (dark yellow sphere, line) CPC and thermomixed at 37 °C for six hours. For perspective, a dashed line at pH = 5.5 indicates where enamel begins to dissolve.

The glycolytic response of the mixed-microbial plaque fluid exposed to the simple aqueous CPC solutions is shown in Figure 2. This is an example plot of characteristic responses to inferior performance (i.e., 0.0% CPC), where the pH drops significantly, or superior performance (e.g., 0.07% or 0.1% CPC), where the pH drops are minimal. The pH of the dissolution of enamel is also included for reference (pH 5.5).

Based on Figure 2 data, Equation (2) was then used to determine the percent change in pH at the six-hour mark. This is referred to as ‘Glycolysis’ in Table 3. Statistical analyses revealed significant differences (*p* < 0.05) exist among the CPC solutions. Significant differences among 0.07%, 0.075% and 0.1% CPC solutions were not observed, though trending reveals the impact of higher CPC content. The best-performing CPC solutions led to minimal drops (e.g., no more than 1% as provided by 0.05% CPC solution), meaning these were the best at inhibiting fermentation of sugar.

### 3.4. Spectrophotometry of QAC Test Strips Immersed in Commercial CPC Mouthwashes

Similar to that performed for simple CPC solutions, quantitative CIELAB color assessments were made on QAC strips immersed in commercial CPC mouthwashes listed in Table 1. The L*, a*, and b* parameters are summarized in Table 4, along with the color change (*ΔE*) relative to the 0 ppm square on the QAC strip as determined from Equation (1). The same L*, a*, and b* values listed above were used again in these calculations.

Relative to immersions in simple CPC solutions, the QAC strips produced distinct coloring details as summarized in Table 3. With respect to L*, the lightness remains relatively constant across the formulations, ranging between 86.60 and 88.70. For the b* parameter, the values shifted further negative relative to the simple 0.1% CPC solution, where all of the values trend towards ‘less yellow and more blue’ on the b* color axis. Finally, the a* parameter continues to be negative but even more relative to simple CPC solutions, further indicating a ‘more green’ profile. The resulting perceived color differences, *ΔE*, are listed in the fifth column. The largest color difference is observed for the ClōSYS^®^ (0.075% CPC) mouthwash, while Smartmouth™ (0.05% CPC) produced the smallest color difference among the CPC-containing mouthwashes. All CPC-containing formulations were significantly different than the negative control (0.0% CPC), and the 0.05% CPC mouthwashes were significantly different relative to either the 0.07% or 0.075% CPC-containing mouthwashes.

### 3.5. Glycolytic Responses from Commercial CPC Mouthwashes

**Figure 3:**
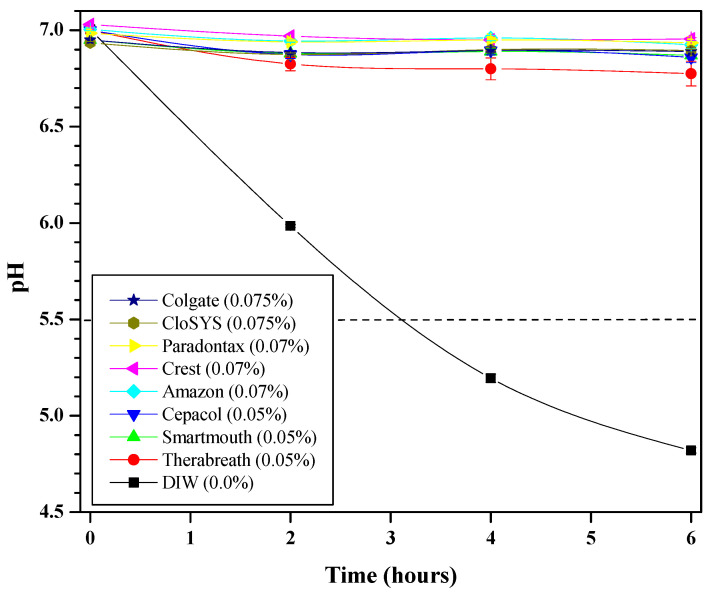
Mean (SD) glycolytic responses (as measured via pH in duplicate) produced from harvested human plaque treated with DIW (0.0% CPC) (black squares, line) or commercial mouthwashes with different CPC content (0.05%, 0.07%, or 0.075% CPC) and thermomixed at 37 °C for six hours. For perspective, a dashed line at pH = 5.5 indicates where enamel begins to dissolve.

Similar to the simple CPC solutions, the glycolytic response of the mixed-microbial plaque fluid exposed to commercial CPC-containing mouthwashes is shown in Figure 3. Again, the inferior performance is marked by the negative control (0.0% CPC solution). In this figure, however, all of the commercial mouthwashes performed well, with Therabreath™ as the least effective group.

Based on Figure 3 data, Equation (2) was then used to determine the percent change in pH at the six-hour mark and is referred to as ‘Glycolysis’ in Table 4. Statistical analyses revealed some significant differences (*p* < 0.05) exist among the commercial mouthwashes: Therabreath™ was the least effective among the 0.05% CPC groups, while all groups were significantly better than the negative control. Though not significant, trends show that higher CPC content formulations tend to provide the best antiglycolysis performance, with the two 0.075% CPC solutions yielding the smallest drops in pH.

### 3.6. Correlations Between ΔE and Glycolysis

**Figure 4:**
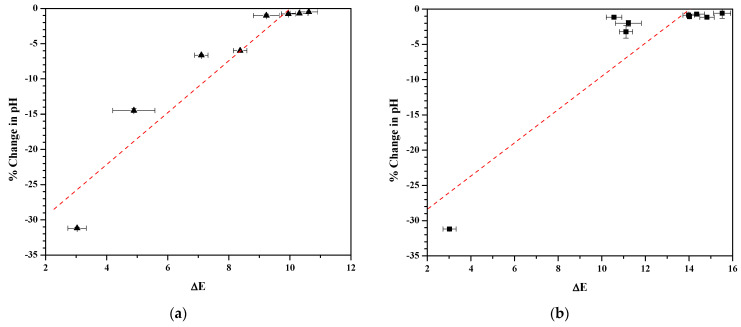
Relationship between color differences (mean *ΔE* ± SD) and glycolysis (mean % Change in pH ± SD) for (**a**) simple CPC solutions corresponding to Table 3 (solid triangles); (**b**) commercial CPC mouthwashes corresponding to Table 4 (solid spheres). Each graph includes a red dashed line denoting linear fits.

Correlations between the color differences (*ΔE*) and glycolysis are shown for simple CPC solutions and commercial CPC mouthwashes in Figure 4. Each figure corresponds to the fifth and sixth columns in Table 3 and Table 4. The linear fit parameters for each of these comparisons are summarized in Table 5, which also includes the results from an additional linear fit for commercial mouthwashes excluding those that are formulated with zinc salts (graph not shown). In each case, the slope, intercept, and linear correlation coefficient (R^2^) are presented. Due to the relatively strong correlations of the linear fits, we then calculated the Pearson correlation coefficient (PCC). The strongest correlations were produced for simple CPC solutions, with the commercial mouthwashes not comprising zinc salts having the next-best correlations. Although also providing a reasonable correlation, the presence of zinc in the CPC mouthwashes produced the smallest coefficients.

## 4. Discussion

In the course of formulating safe and effective CPC-containing mouthwashes, there is a strong need to identify tools that can efficiently estimate product performance. The use of QAC strips, which have been in use for many years to determine levels of quaternary compounds in several fields where effective sanitizers or disinfectants are important [12,13,14,15,16,17], have not been applied to the evaluation of CPC-based oral care formulations. To determine if the QAC strips have any potential use for formulators and brands in the development or testing of CPC-based mouthwashes, it was important to correlate results from these strips to those generated from a separate proven methodology. Glycolysis has previously been shown to correlate very well with the cellulose technique [3,4]. Moreover, industry-wide, glycolysis is easier to implement technologically and provides direct information on how a given formula performs against plaque constituents. Based on the present results, strong correlations were found between bioavailable CPC determined from the QAC test strips and glycolysis efficacy, thereby the null hypothesis is rejected. This research is among the first, if not the first, to report on the correlation of QAC test strip results to glycolysis as it pertains to CPC-based oral care formulations.

The use of the spectrophotometer is not new in oral care research; in fact, it is a core tool used in the assessment of whitening or staining of tooth enamel [21,23]. Here, we applied the familiar tool to create quantitative parameters that could then be further assessed. Trending and statistical differences demonstrate the sensitivity of using the spectrophotometer to assess these visual-based QAC strips. We note that future assessments might include more-sensitive QAC strips or increased replicates, but the focus here was to determine if CPC content can relate, at least directionally, to color changes obtained from typical off-the-shelf QAC test strips. Furthermore, could those color changes be correlated to glycolytic data? In both cases, strong correlations were found to exist.

As mentioned in the introduction, one of the reasons this is important is that a traditional approach of measuring CPC availability relies on a cellulose technique that either is not widely available or has limitations due to CPC structures (e.g., monomers, micelles, vesicles, etc.) or content (e.g., having at least 0.07% CPC). The cellulose disc technique relies on the negatively charged cellulose component to primarily attract the monomer-based positively charged cetylpyridinium component [3,4,5]. In one respect, the QAC strips utilize similar chemistry, as the negatively charged chromophore reacts with available cetylpyridinium [12,13,14,15]. But in contrast to the cellulose disc method, QAC test strips are non-specialized, widely accessible, and routinely used to assess potency of antiseptic or cleaning agents where microbial contamination is of concern [12,13,14]; importantly, the QAC strips may not be limited to the nature of CPC structures. From a formulation perspective, QAC methods might be preferable due to low costs, ease of use, and rapid results. In fact, as formulators who have created CPC-containing mouthwashes for commercial purposes, we readily embrace the use of QAC strips for these very reasons.

There are several potential implications for using QAC strips as a possible alternative to the cellulosic method. Firstly, the ability to have multiple researchers, formulators, etc., use essentially the same measurement tool helps to provide a consistent source for measurement. Unlike the cellulosic method, which manifests inherent variations due to differences in centrifuges, cellulosic materials, UV detection apparatus, etc., the QAC test strip is fabricated and produced with defined parameters, with the ability of several independent laboratories capable of then using essentially the same QAC test strip. Secondly, not only do QAC test strips provide for a more streamlined approach due to inherently less apparatus, in turn it might also help reduce potential errors, including experimental and operator errors. Finally, these strips may assist formulators, researchers, quality control departments, etc., that do not have access to or knowledge of the cellulosic method to be able to participate in assessments of CPC-based formulations. This is of prime importance, since there are those who may have a strong interest in creating, optimizing, assessing, or even understanding the extent or limitations to how certain CPC-based formulations may work. And, contributing to the development and use of the cellulosic method was the original combination of both CPC and alcohol (e.g., Cēpacol^®^) [2], with the latter providing not only germicidal benefits but, critically, also limiting higher-order CPC geometries due to its renowned emulsifying properties. Because present formulations have largely moved away from the use of alcohol, higher-order CPC structures certainly exist (the nature of which will depend on the given formulation), and this is why using the cellulosic method may underestimate bioactive CPC [5].

But, we recognize there are limitations of the QAC test strip. For instance, at higher CPC content (e.g., exceeding 0.07% CPC), both *ΔE* and glycolysis data appear to taper, indicating possible limits for these assessments. However, this might be less a flaw in the methodologies than the potency of the given treatment. The color-based methods to determine concentration are limited to the buffering range and composition manifest in the testing strip. The QAC test strips used in the present research were not designed to resolve 50 ppm (or less) differences between the 0.05% and 0.1% QAC levels. A more sensitive QAC test strip might readily distinguish the differences within this range and this is something we will certainly explore in future studies.

Separately, the lack of significant differences in glycolysis between 0.07% and 0.1% CPC in this evaluation mirrors the lack of significant differences in clinical outcomes [24]; thus, the lack of significant differences among 0.07%, 0.075%, and 0.1% CPC is not necessarily unexpected and remains consistent with our prior observations [11]. Any observation of significant differences between two similar high-level CPC mouthwashes, such as 0.075% CPC for Colgate^®^ and ClōSYS^®^, will likely be elusive (as observed here), unless the formulation has a manifest flaw resulting in diminished bioavailability of CPC, as discussed below.

As an example of a poorly performing CPC-containing mouthwash, we also examined a now-discontinued CPC-based mouthwash (BreathRx, Philips Sonicare, Bothell, WA, USA) which had a labeled 0.075% CPC content. Through evaluation with the same QAC strips used in this report, we found this formulation had less than 400 ppm available CPC (or, less than 0.04% CPC). This impaired level of CPC was reflected in its poor glycolysis performance: the percent change in pH at six hours was −6.5%, a level that was almost twice as less than the least performing CPC mouthwash group tested here (i.e., 0.05% CPC Therabreath™ mouthwash, whose percent drop in pH was −3.21%) and comparable to that obtained from the simple 0.04% CPC solution (i.e., percent drop in pH was −5.99%). Because impaired CPC availability is directly related to glycolysis [3,4], and does not meet the minimum bioavailable limit of CPC needed to meet the FDA antiplaque/antigingivitis monograph [1], it is highly unlikely the formulation would compare favorably if tested clinically against a properly formulated CPC mouthwash, such as the Crest^®^, Paradontax or Colgate^®^ mouthwashes evaluated in this work.

Differences between the simple CPC solutions and the commercial mouthwashes demonstrate the presence of other ingredients in the commercial mouthwash contribute to the observed color. In particular, we note that two of the formulations contained zinc salts (zinc lactate in Colgate^®^ and zinc chloride in Smartmouth™), which appeared to have influenced the QAC strip color (as assessed via the spectrophotometer). We reason that the positively charged Zn^2+^ species likely complexed, even minimally, with the negatively charged chromophore in the QAC strip, thus, occupying sites theoretically reserved for cetylpyridinium. In both cases, b*, and therefore *ΔE*, were somewhat reduced relative to other groups at the same CPC level that lacked the zinc salts. Notably, Zn^2+^ is known to influence microbial activity [25,26,27,28], a fact we have also observed in our prior glycolysis evaluations [11]. This is likely one reason the Colgate^®^ formulation exhibited directionally higher glycolysis benefits relative to the similar 0.075% CPC content of the ClōSYS^®^ mouthwash. The fact that *ΔE* was higher for ClōSYS^®^ than Colgate^®^ suggests the lack of zinc salts allowed maximum interaction between CPC and the QAC test strip chromophore.

The observation that ClōSYS^®^ produced the greatest color change (*ΔE*) speaks to its absence of any surfactant (e.g., PEG-40 castor oil or poloxamer) that might otherwise possibly influence CPC availability [3,4]. This comes despite the presence of trisodium phosphate, which could suggest this salt presents minimal issues with CPC that would appear in either the QAC test strip or glycolysis assays. In fact, ClōSYS^®^ was nearly as ‘simple’ as the Therabreath™ mouthwash in terms of ingredients utilized, though the latter used a surfactant (poloxamer 407), which may have reduced its glycolysis potency: those polymeric systems with hydrophilic or lipophilic activity are known to affect CPC structure [3,7,8]. Though several of the other mouthwashes also comprise poloxamers, it is possible the formulation recipes were sufficiently optimized to minimize undesirable CPC–poloxamer interactions. While Cēpacol^®^ used some phosphate salts and edetate disodium, its alcohol content may have limited effects from these ingredients on CPC structure. With respect to Smartmouth™, this formulation delivered potent glycolysis benefits at the 0.05% CPC level, but this may be attributed in part to both sodium chlorite and zinc chloride, as this formulation also had the smallest color change among the 0.05% CPC mouthwashes (i.e., meaning CPC structure could certainly have been affected).

As a final consideration, it may be possible that the dye used in a given mouthwash may influence the QAC test strip. As manufacturers typically do not state the dye level used, we can infer based on our experience as well as available information in the literature. For those formulations with a bluish hue (which represent five of the eight tested), we expect the blue color to be no greater than 0.01% due to its color potency. At this level, one would expect minimal contributions to the QAC dye strip based on the example color level shown in Table 1 (~0.08%). Of the remaining mouthwashes, one used yellow dye, which was also the one that used alcohol (Cēpacol^®^), and we estimate a comparable level. The remaining two did not use dyes, so interference would be moot.

## 5. Conclusions

In conclusion, while the cellulose-disc method remains a trusted option, it relies on a strong CPC dipole (i.e., predominant monomer-like structure), which may not always be dominant in a given mouthwash formulation. In contrast, for the first time we have shown that QAC test strips might allow for screening and research activities due to possible allowances of a broader set of CPC structures (e.g., micelles, vesicles, etc.). Though direct comparison between these two methods along with CPC structural evaluations are reserved for a future study, from a formulation perspective, QAC test strips might be preferable due to the relatively low cost, ease of use, and rapid results. Most importantly, the glycolysis results obtained here appear to correlate very well with QAC test strip outcomes for a variety of CPC formulations. These observations may help better understand the mechanism of action of a formulation, as well as identify possible formulary pitfalls or synergies. In fact, as formulators who have created CPC-containing mouthwashes for commercial purposes, we readily embrace the use of QAC strips for these very reasons. Therefore, based on our evaluations and data presented herein, we believe the use of QAC test strips could become an essential tool in the development and evaluation of CPC-containing oral care formulations. Future directions will explore more sensitive QAC strips as well as influences of other ingredients on CPC availability and glycolysis.

## Figures and Tables

**Table 1:** Example of recipe (pH ~ 3) used to detect colors changes due to QAC [15].

Ingredient	Percent by Weight
Bromophenol blue	0.08
Sodium acetate	12.50
Glacial acetic acid	62.50
Distilled water	24.92
Total	100.00

**Table 2:** Details of commercial CPC mouthwashes used in this study.

Mouthwash	Wt. % CPC	pH	Other Ingredients
Colgate^®^ Total (Colgate-Palmolive Co., New York, NY, USA)	0.075%	4.4	Water, glycerin, propylene glycol, sorbitol, poloxamer 407, zinc lactate, flavor, potassium sorbate, lactic acid, sodium saccharin, sucralose, blue 1
ClōSYS^®^ Healthy Gums (Rowpar Pharmaceuticals, Bridgewater, NJ, USA)	0.075%	5.2	Purified water, trisodium phosphate, citric acid, potassium sorbate, sodium benzoate, mint flavor, sucralose
Paradontax Active Gum Health (GSK Consumer Healthcare, Warren, NJ, USA)	0.07%	3.8	Water, glycerin, flavor, poloxamer 188, sodium saccharin, propylene glycol, sodium benzoate, sucralose, benzoic acid, blue 1
Crest^®^ Pro Health Clean Mint (Procter & Gamble, Cincinnati, OH, USA)	0.07%	3.8	Water, glycerin, flavor, poloxamer 407, sodium saccharin, methylparaben, sucralose, propylparaben, blue 1
Amazon basics (Amazon.com Services, Seattle, WA, USA)	0.07%	3.7	Water, glycerin, flavor, poloxamer 188, sodium saccharin, propylene glycol, sodium benzoate, sucralose, benzoic acid, blue 1
Smartmouth™ Clinical (Triumph Pharmaceuticals, St. Louis, MO, USA)	0.05%	5.1	Solution 1: Purified water, sodium benzoate, sodium chlorite, benzoic acid Solution 2: Purified water, glycerin, poloxamer 407, propylene glycol, poloxamer 124, zinc chloride, flavor, sodium benzoate, benzoic acid, sodium saccharin, sodium chloride, benzyl alcohol, D&C Yellow No. 10, FD&C Blue No. 1
Therabreath™ Healthy Gums (Church & Dwight, Ewing, NJ, USA)	0.05%	6.1	Water, glycerin, poloxamer 407, flavor, sucralose
Cēpacol^®^ Antibacterial (Reckitt Benckiser, Parsippany, NJ, USA)	0.05%	7.4	Purified water, alcohol 14% v/v, glycerin, sodium phosphate dibasic, eucalyptus oil, polysorbate 80, methyl salicylate, cinnamon oil, peppermint oil, sodium saccharin, sodium phosphate monobasic anhydrous, menthol, edetate disodium, FD&C Yellow No. 5

**Table 3:** Spectrophotometric and glycolysis summary for simple aqueous CPC solutions.

Wt. % CPC	L*	a*	b*	*ΔE* ^1^	Glycolysis ^2^
0.10%	82.86 (0.10)	−2.38 (0.06)	1.28 (0.31)	10.62 (0.29) ^A^	−0.50 (0.10) ^a^
0.075%	82.93 (0.11)	−2.78 (0.07)	1.73 (0.30)	10.31 (0.28) ^B^	−0.71 (0.00) ^a,b^
0.07%	83.09 (0.07)	−2.77 (0.01)	2.08 (0.25)	9.95 (0.23) ^C^	−0.78 (0.10) ^a,b^
0.05%	83.37 (0.20)	−2.99 (0.11)	2.87 (0.40)	9.23 (0.42) ^D^	−1.00 (0.20) ^b^
0.04%	83.84 (0.21)	−3.11 (0.05)	3.70 (0.15)	8.37 (0.22) ^E^	−5.99 (0.00) ^c^
0.03%	84.46 (0.18)	−3.07 (0.09)	4.97 (0.24)	7.10 (0.22) ^F^	−6.63 (0.10) ^d^
0.02%	85.67 (0.40)	−2.72 (0.17)	7.14 (0.69)	4.89 (0.69) ^F^	−14.48 (0.30) ^e^
0.00%	86.60 (0.27)	−1.36 (0.08)	9.25 (0.35)	3.03 (0.30) ^F^	−31.19 (0.13) ^f^

^1^ Uppercase letter superscripts (A, B, etc.) for Mean (SD) *ΔE* calculated from L*, a*, and b* from Equation (1) denote significant differences (*p* < 0.05) with A > B, etc., within this column. ^2^ Lowercase letter superscripts (a, b, etc.) for Mean (SD) Glycolysis (% Change in pH) data from Equation (2) denote significant differences (*p* < 0.05) with a > b, etc., within this column.

**Table 4:** Spectrophotometric and glycolysis summary for commercial CPC mouthwashes listed in Table 1.

Mouthwash	L*	a*	b*	*ΔE* ^1^	Glycolysis ^2^
Colgate^®^	86.95 (0.10)	−4.29 (0.17)	−3.91 (0.27)	13.98 (0.26) ^B^	−0.58 (0.00) ^a^
ClōSYS^®^	86.11 (0.17)	−3.36 (0.04)	−5.55 (0.37)	15.52 (0.39) ^A^	−0.72 (0.20) ^a^
Paradontax	86.72 (0.60)	−3.73 (0.22)	−4.39 (0.29)	14.36 (0.36) ^B,C^	−0.86 (0.00) ^a,b^
Crest^®^	87.28 (0.01)	−3.79 (0.01)	−4.14 (0.00)	14.02 (0.00) ^B^	−1.07 (0.10) ^a,b^
Amazon	86.51 (0.23)	−3.76 (0.06)	−4.83 (0.30)	14.82 (0.33) ^C^	−1.14 (0.20) ^a,b^
Smartmouth™	88.70 (0.35)	−4.28 (0.06)	−0.52 (0.34)	10.52 (0.36) ^D^	−1.15 (0.00) ^a,b^
Cēpacol^®^	88.24 (0.27)	−4.05 (0.08)	−1.29 (0.58)	11.22 (0.59) ^D^	−2.00 (0.40) ^b^
Therabreath™	88.56 (0.44)	−4.42 (0.03)	−1.09 (0.28)	11.11 (0.30) ^D^	−3.21 (0.91) ^c^
DIW	86.60 (0.27)	−1.36 (0.08)	9.25 (0.35)	3.03 (0.30) ^E^	−31.19 (0.13) ^d^

^1^ Uppercase letter superscripts (A, B, etc.) for Mean (SD) ΔE calculated from L*, a*, and b* from Equation (1) denote significant differences (*p* < 0.05) with A > B, etc. within this column. ^2^ Lowercase letter superscripts (a, b, etc.) for Mean (SD) Glycolysis (% Change in pH) data from Equation (2) denote significant differences (*p* < 0.05) with a > b, etc. within this column.

**Table 5:** Linear fitting parameters and coefficients of color and glycolysis data from Table 2 and Table 3.

Group	Slope	Intercept	R^2^	PCC *
^1^ CPC Solutions	3.68	−36.86	0.90	0.95
^2^ Commercial MW w/o Zn	2.46	−35.25	0.89	0.94
^3^ All Commercial MW	2.36	−33.10	0.82	0.91

* Pearson correlation coefficient (PCC) for the correlation between *ΔE* and Glycolysis. ^1^ Linear fit of data from simple CPC solutions listed in Table 3. ^2^ Linear fit of data from commercial CPC MW solutions listed in Table 4 not including Colgate^®^ Total and Smartmouth™. ^3^ Linear fit of data from all commercial CPC MW solutions listed in Table 4.

## Data Availability

Data presented in this paper are available upon written request.

## Abbreviations

The following abbreviations are used in this manuscript:

US	United States
FDA	Food & Drug Administration
NMR	Nuclear Magnetic Resonance
QAC	Quaternary ammonium compound
CPC	Cetylpyridinium chloride
OTC	Over-the-Counter
MW	Mouthwash
HPLC	High performance liquid chromatography
UV	Ultraviolet
TSB	Tryptic soy broth
CIE	International Commission on Illumination
LAB	*L* a* b**
ANOVA	Analysis of variance
SNK	Student-Newman-Keuls
PCC	Pearson correlation coefficient
